# Comparative effectiveness of electroacupuncture VS neuromuscular electrical stimulation in the treatment of chronic low back pain in active-duty personals: A single-center, randomized control study

**DOI:** 10.3389/fneur.2022.945210

**Published:** 2022-09-13

**Authors:** Xiao-yan Meng, Lan Bu, Jia-ying Chen, Qiu-jia Liu, Li Sun, Xiao-long Li, Fei-xiang Wu

**Affiliations:** ^1^Department of Critical Care Medicine, Eastern Hepatobiliary Surgery Hospital, Navel Medical University, Shanghai, China; ^2^Department of Anesthesiology and Pain Center, Shanghai Changhai Hospital, Navel Medical University, Shanghai, China; ^3^Department of Anesthesiology, Eastern Hepatobiliary Surgery Hospital, Navel Medical University, Shanghai, China; ^4^Department of Traditional Chinese Medicine, Shanghai Changhai Hospital, Navel Medical University, Shanghai, China; ^5^Department of Spinal Surgery, Shanghai Changhai Hospital, Navel Medical University, Shanghai, China

**Keywords:** neuromuscular electrical stimulation, chronic low back pain, military service, electroacupuncture, randomized control study

## Abstract

**Introduction:**

Low back pain (LBP) is the most prevalent form of chronic pain in active-duty military personnel worldwide. Electroacupuncture (EA) and neuromuscular electrical stimulation (NMES) are the two most widely used treatment methods in the military, while evidence for their benefits is lacking. The aim of this randomized clinical trial is to investigate the effectiveness of EA vs. NMES in reducing pain intensity among active-duty navy personals with chronic LBP.

**Methods:**

The study is designed as a single-center, randomized controlled trial. The primary outcome is a positive categorical response for treatment success in the first-time follow-up, which is predesignated as a two-point or greater decrease in the NRS score and combined with a score > 3 on the treatment satisfaction scale. The secondary outcomes include pain intensity, rate of treatment success, and Oswestry Disability Index (ODI) fear-avoidance beliefs questionnaire (FABQ) score along with muscular performance. The first follow-up starts on the first day after completing the last treatment session, and then the 4-weeks and 12-weeks follow-up are applied *via* telephone visit.

**Results:**

Eighty-five subjects complete the treatment diagram and are included in the analysis. For the primary outcome, no difference has been found between EA and NMES, with 65.1% (28 in 43) individuals reporting a positive response to EA treatment, while 53.5% (23 in 43) in NMES. However, for longer follow-ups, superiority in positive response of EA has been found in 4-weeks (26 in 39, 66.7% vs. 16 in 40, 40%; *P* = 0.018) and 12-weeks (24 in 36, 66.7% vs. 12 in 36, 33.3%; *P* = 0.005) follow-up. In the regression analysis, baseline pain intensity and FABQ score are identified to be highly associated with positive treatment outcomes. Finally, the subgroup analysis suggests that EA treatment is associated with better long-term outcomes in patients with LBP with a severe pain score (NRS score >4, Figure 4B) and stronger fear-avoidance beliefs.

**Conclusion:**

Both the EA and NMES are associated with a positive response in treating military LBP, and the former offers lasting benefits in the later follow-ups. Thus, electroacupuncture is a more recommended treatment for military LBP. A lot of research is needed to verify an efficient and standardized treatment session, with more information and evidence about indications for these treatments.

**Trial registration:**

ChiCTR, (ChiCTR2100043726); registered February 27, 2021.

## Introduction

Low back pain (LBP) is the most prevalent form of chronic pain in the military worldwide (1). According to previous studies, LBP affects over 150,000 active-duty soldiers annually in the United States army and occupies around 7% of all medical visits in the US and UK military (2, 3). Medical reports of the War of Iraq and Afghanistan also indicate that LBP accounts for 18% of non-battle injury evacuations (4–6). More importantly, LBP becomes chronic in about 20–30% of those afflicted. Chronic LBP is often combined with severe functional limitations and psychological symptoms, leading to increased disability and reduced quality of life (7). The reported mobility of LBP in the Chinese army varies a lot, with a prevalence of 4.7–15% in certain types of troops.

Previously, numerous studies have explored the risk factors and prevention strategies for military LBP, from the Prevention of Low Back Pain in the Military (POLM) trials in 2007 to the very recent Resolving the Burden of Low Back Pain in Military Service Members and Veterans (RESOLVE) and Sequential Multiple-Assignment Randomized Trial (SMART) programs (8, 9). According to these results, prior history of LBP, previous musculoskeletal injury, less time in physical training, female sex, and lower rank were consistent risk factors for LBP in active-duty personnel, noting that these risk factors are significantly different from those of the general population and even for veterans (10). Besides, some types of armies are prone to have higher risks for LBP, including pilot, infantry, and driver (8, 11). Yet, the study of the naval population is extremely rare, which may be because data collection from on-duty naval officers and soldiers is difficult.

Currently, there has been no reliable treatment for LBP. The investigation of risk factors and prevention strategies for LBP remains to be a research priority (12–14). Practically, pharmacological or interventional treatments may be subject to certain conditions and are associated with severe adverse effects, such as addiction and surgical complications. Thus, interests in non–pharmacological treatments (NPT) for LBP have long been a research priority, especially in military populations. Previously, Dr. George et al. (15, 16) have launched a cluster of the Prevention of Low Back Pain in the Military (POLM) trials to determine the effectiveness of core stabilization exercises and education programs in preventing LBP. A lot of research highlights the idea that active treatment in the early episode of LBP is vital before chronic disability symptoms occur. However, the application of these active treatments may also exacerbate pain and reduce voluntary muscle (14), especially for active-duty personals, as they may have strict training and working diagrams. Thus, a lot of research for effective NPTs is needed.

Among numerous treatments for chronic LBP, non–invasive electrotherapies of electroacupuncture (EA) and neuromuscular electrical stimulation (NMES) are proven to be feasible and effective (17–19). The application of acupuncture for pain management has a history of thousands of years in China, while NMES is an emerging treatment conception and promotes promptly in the last decades. Both of these two techniques are well-received in naval populations, as they can be applied during navigation and training days. Since the electric current produced by NMES is superficial, the effect of NMES on muscle contraction is more easily affected by the size and position of electrodes. Therefore, EA is supposed to produce better results in muscle strengthening and reduction of discomfort (14). However, several recent trials report no superiority of EA to sham acupuncture or transcutaneous simulation in treating chronic pain, yet both EA and NMES have not been rigorously tested in clinical trials involving active-duty soldiers (17). Based on previous trials and our experience, we hypothesize that while both EA and NMES alleviate LBP, active-duty navy personals may benefit more from EA in pain-relieving and LBP recurrence. The aim of this randomized clinical trial is to investigate the effectiveness of EA vs. NMES in reducing pain intensity among active-duty soldiers with chronic LBP (cLBP), and with the additional aim of identifying outcome predictors in a pragmatic setting.

## Methods

### Study design and participants

The study is designed as a single-center, randomized controlled trial. The protocol is in accordance with the Standard Protocol Items: Recommendations for Interventional Trials (SPIRIT) statement. This trial is registered at Chictr.org.cn with ID number ChiCTR2100043726. The study is approved by the Local Ethics and Security Committee (EHBHKY2020-K-058), and written informed consent was obtained from all participants.

The main inclusion criteria are navels aged 18 to 35 years, with reported non-specific low back pain for at least 3 months (in accordance with the definition of cLBP) (20), and have an average back pain intensity of at least 3 on a 0 to 10 numerical rating scale in the previous week. The main exclusion criteria include (1) back pain with specific causes of disc compression or spinal stenosis, which are diagnosed by examination or magnetic resonance imaging; (2) combined with other severe pain conditions; (3) having accepted acupuncture or physiotherapy in the last 3 months; and (4) cannot accept regular treatment due to their own training schedule. Recruitment occurred from January 2021 to March 2021, and we have conducted a questionnaire survey (Supplementary Table 1) for all soldiers in the troop ahead of this trial for preliminary screening of participants.

### Blinding, randomization, and sample size calculation

This study is performed as a single-blinded study. Given the nature of the intervention, blinding of therapists and patients is not possible; however, investigators responsible for data collection and data analysis will be blinded to the study arms. Randomization is conducted using a computerized random number generator, and participants will be given a randomization number and will be allocated according to indications inside that numbered envelope. For sample size calculation, we assume a 40% of treatment success in the NMES group and 30% higher in the EA group according to the previous studies (18, 21). With 80% power and two-tailed α = 0.05, the estimated sample size should be 40 for each group by calculation *via* PASS 11 (Utah, United States). Considering the potential loss of follow-up cases in military populations, we recruited 46 subjects in each group.

### Interventions

All participants accept a brief education of basic knowledge of LBP prior to treatment. EA treatments are performed by an experienced therapist, and the subjects are positioned in the prone position. After skin degerming, acupuncture needles are inserted into the skin of bilateral Jia Ji (M-BW-35) acupoints (L3-S1) for about 5 cm, according to the World Health Organization Standardized Acupuncture Point Location. The electric apparatus was applied to the acupoints with a dilatational wave using a 50 Hz frequency and a comfortably tolerated maximum current intensity. For NMES, two pairs of 40 × 40 mm electrodes were placed at the same acupoints to the EA, and the current intensity was gradually increased to the maximum tolerance of participants. Each participant was treated for a total of 30 min each session, 6–7 times in 2 weeks. Non-steroidal anti-inflammatory drugs are permitted as rescue medications only when patients report deteriorated unbearable pain during treatment, and cointerventions between treatment and follow-up visits are not permitted.

### Data collection and follow-up

Baseline data collected include demographic information, type of work, concomitant pain conditions, type of analgesic or treatment history, smoking status, average back, and leg pain score on a 0–10 numerical rating scale (NRS) over the past week. Psychological and physiological factors are estimated with the Oswestry Disability Index version 2 (ODI v.2.0) score (22) and fear-avoidance beliefs questionnaire (FABQ) score (23). Besides, functions of multifidus are estimated by ultrasound scanning (SonoSite, United States), and the recorded data include thickness and cross-sectional area (CSA, cm2) at rest and contraction state of both sides, at the L4 level (24, 25). Calculated muscle functions for analysis include multifidus symmetry, contraction rate, and average CSA. The symmetry of multifidus is defined as the proportional difference of relatively larger side to smaller side at rest state, and contraction of multifidus is defined as the proportional difference of mean thickness at contraction to rest state. These variables have been reported to be associated with LBP. Detailed measurement and calculation methods of muscles are according to the previous studies and are described in Supplementary Table 2.

The first follow-up starts on the first day after completing the last treatment session, and data recorded include NRS, ODI, FABQ score, a 5-point Likert scale measuring treatment satisfaction (1 = very unsatisfied, 2 = unsatisfied, 3 = neither satisfied nor unsatisfied, 4 = satisfied and 5 = very satisfied), as well as the functions of multifidus measured by ultrasound. Then the 4-weeks and 12-weeks follow-up are applied *via* telephone visit, to record the average NRS score in the past week and the Likert satisfaction scale.

### Outcomes

The primary outcome measure is a change in average pain score (NRS) and treatment satisfaction scale (Likert), and the primary end point is 1 day after the treatment diagram, and 4-weeks and 12-weeks follow-up. A positive categorical response to the treatment is defined as a two-point or greater decrease in the NRS score and a score of > 3 on the Likert scale, according to previous studies (26). Thus, a positive rate for treatment is then achieved in each group, and changes in NRS scores are also calculated. At each follow-up, along with pain scores, secondary outcome measures recorded included ODI and FABQ scores along with muscular performance *via* ultrasound imaging in 1 day after the last treatment session.

### Statistical analyses

All statistical analyses were performed with SPSS 25.0 (IBM SPSS Statistics, Armonk, New York). We performed a complete-case analysis on our primary outcomes according to the intention-to-treat (ITT) principle. The normality of all datasets was tested using Kolmogorov–Smirnov tests. Continuous variables were presented as the mean ± standard deviation (std) or median with IQR (interquartile range), while categorical variables were presented as numbers and proportion (%). Multiple imputation method is used for missing values. In comparing baseline characters and multifidus imaging outcomes between treatment groups, Student's *t*–test, the Chi-square test, or the Wilcoxon test is applied accordingly. For outcomes, the categorical treatment responses between groups were compared using the Chi-square test, and changes in NRS values were compared with the Wilcoxon test. To explore factors associated with positive treatment responses in all follow-ups, a univariate logistic regression model for all variables potentially associated with outcomes has been applied, and then, all variables with P<0.1 in the univariate analysis are further adjusted into multivariable analysis with “input” selection model. Besides, we also applied univariate and multivariate linear regression analyses for the continuous outcome (change in NRS scores after treatment compared with baseline NRS). Further, we also applied subgroup analysis to different baseline pain intensities and different baseline FABQ scores.

A *P*-value < 0.05 at two tails is considered statistically significant.

## Results

**Figure 1:**
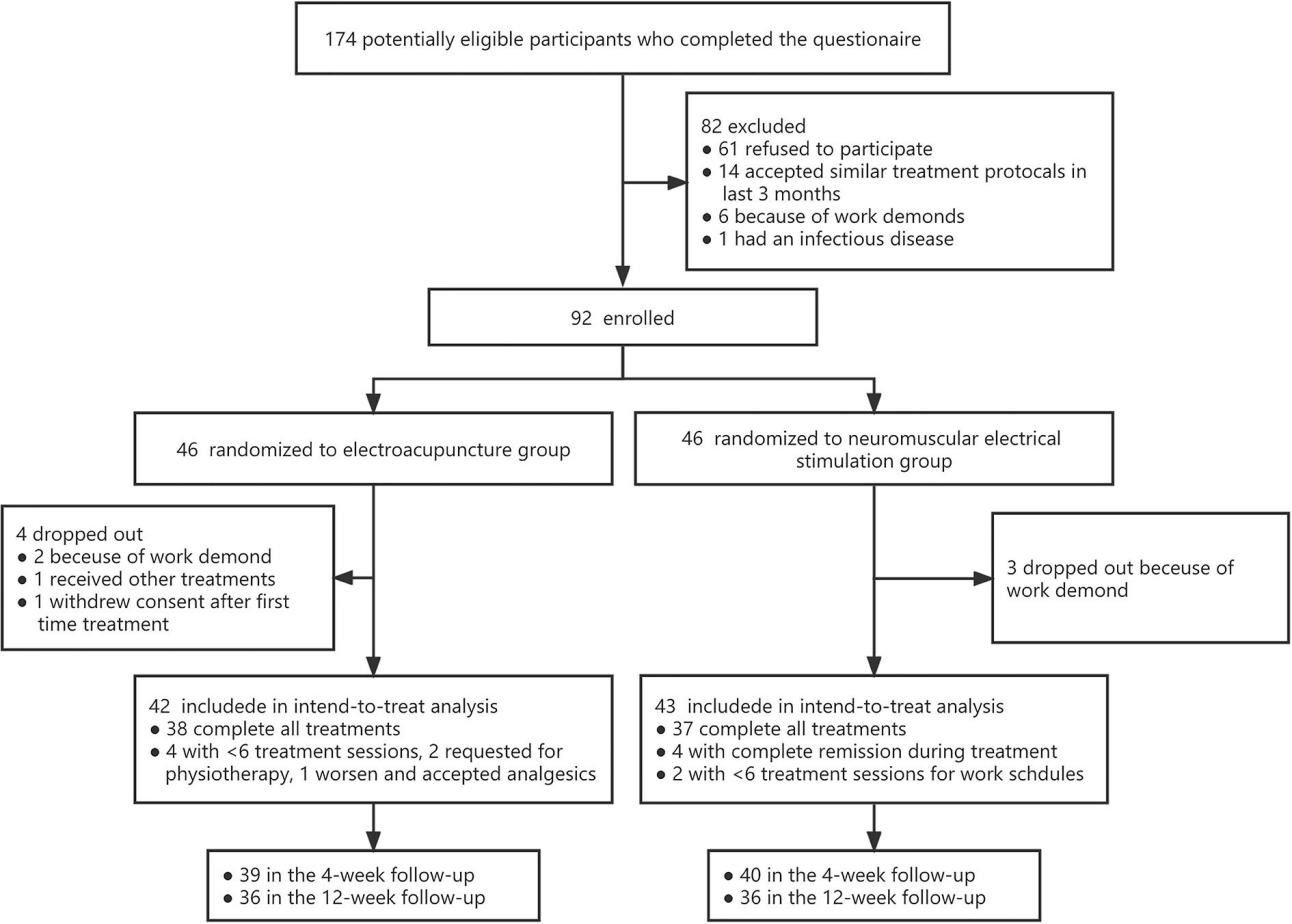
Study flow.

A total of 92 patients are recruited and equally randomized to the EA and NMES groups, and 42 subjects in the EA group complete the treatment and attend the assessment visit and are recruited into the ITT analysis. Comparatively, 43 individuals in the NMES group remained in the ITT analysis. The study flow is shown in Figure 1. In general, enrolled subjects are all male soldiers at a young age (26.5 ± 4.6), and the baseline NRS score for them is 4.5 ± 1.5, while 42 (45.7%) of them suffered moderate to severe LBP syndrome (with an NRS score > 4); 43.5% of them have LBP for more than 1 year and 29.3% of them have actively sought for medical treatment previously. The median (IQR) score for ODI and FABQ is 20 (13.3, 33.5) and 21 (15.25, 30), respectively. No significant differences in baseline characters between the two groups, as shown in Table 1.

**Table 1:** Baseline characters.

**Variable**	**General** ** (*N* = 92)**	**EA** ** (*N* = 46)**	**NMES** ** (*N* = 46)**	***P* value**
Baseline VAS (mean ± sd)	4.5 ± 1.5	4.7 ± 1.5	4.4 ± 1.5	0.264
Baseline VAS (moderate to severe pain, *n*, %)	42 (45.7)	19 (41.3)	23 (50.0)	0.402
Age (year, mean ± sd)	26.5 ± 4.6	26.2 ± 4.1	26.9 ± 5.0	0.454
BMI (mean ± sd)	22.7 ± 3.1	23.3 ± 2.1	22.2 ± 3.9	0.088
Education background (postgraduate, *n*, %)	17 (19.5)	5 (10.9)	12 (26.1)	0.142
Serving time (*n*, %) < 5 years 5–10 years >10 years	23 (25) 42 (45.7) 26 (28.2)	11 (23.9) 22 (47.8) 13 (28.3)	12 (26.7) 20 (44.4) 13 (28.9)	0.737
Smoking history (*n*, %) none 0–10 years >10 years	41 (44.5) 34 (36.9) 17 (18.5)	24 (52.2) 14 (30.4) 8 (17.4)	17 (37.0) 20 (43.5) 9 (91.6)	0.315
Heavy physical demand (*n*, %)	75 (81.5)	36 (78.3)	39 (84.8)	0.418
History of injury (n, %)	33 (35.8)	15 (32.6)	18 (39.1)	0.514
Job type (*n*, %)	64 (69.6)	28 (60.9)	36 (78.3)	0.088
History of treatment for LBP (*n*, %)	27 (29.3)	18 (39.2)	9 (19.6)	0.095
Time of LBP (*n*, %)	40 (43.5)	22 (47.9)	18 (39.1)	0.463
Baseline ODI score (IQR)	20 (13.3, 33.5)	17.7 (12.2, 33.3)	22.2 (14.4, 37.3)	0.122
Baseline FABQ score (IQR)	21 (15.25, 30)	19 (14, 24.8)	24 (16, 37)	0.097

EA, electroacupuncture; NMES, neuromuscular electrical stimulation; sd, standard deviation; LBP, low back pain; ODI, The Oswestry Disability Index; FABQ, fear-avoidance beliefs questionnaire.

Functions of multifidus before and after treatment are shown in Table 2. Although the muscular performance between the two treatment groups remains incomparable due to the heteroskedasticity of data, we have identified a significant difference in symmetry and construction rate of multifidus after treatment in both of the two groups.

**Table 2:** Imaging outcomes for multifidus.

	**General**	**EA**	**NMES**
**Baseline**			
Symmetry (%)	0.104 (0.061, 0.17)	0.101 (0.067, 0.169)	0.112 (0.052, 0.174)
Construction (%)	0.413 (0.315, 0.521)	0.413 (0.264, 0.503)	0.411 (0.322, 0.58)
CSA (cm^2^)	7.005 (5.126, 8.276)	7.248 (5.484, 8.625)	6.390 (4.570, 7.765)
**After treatment**			
Symmetry (%)	0.135 (0.062, 0.216)^*^	0.147 (0.089, 0.27)^*^	0.134 (0.044, 0.196)
Construction (%)	0.483 (0.296, 0.624)^*^	0.444 (0.273, 0.627)^*^	0.499 (0.358, 0.605)^*^
CSA (cm^2^)	7.118 (5.464, 8.493)	7.720 (5.543, 8.551)	6.740 (5.420, 8.590)

* P <0.05 in paired Wilcoxon test. EA, electroacupuncture; NMES, neuromuscular electrical stimulation; CSA, cross-sectional area. Data are presented as median (IQR).

**Figure 2:**
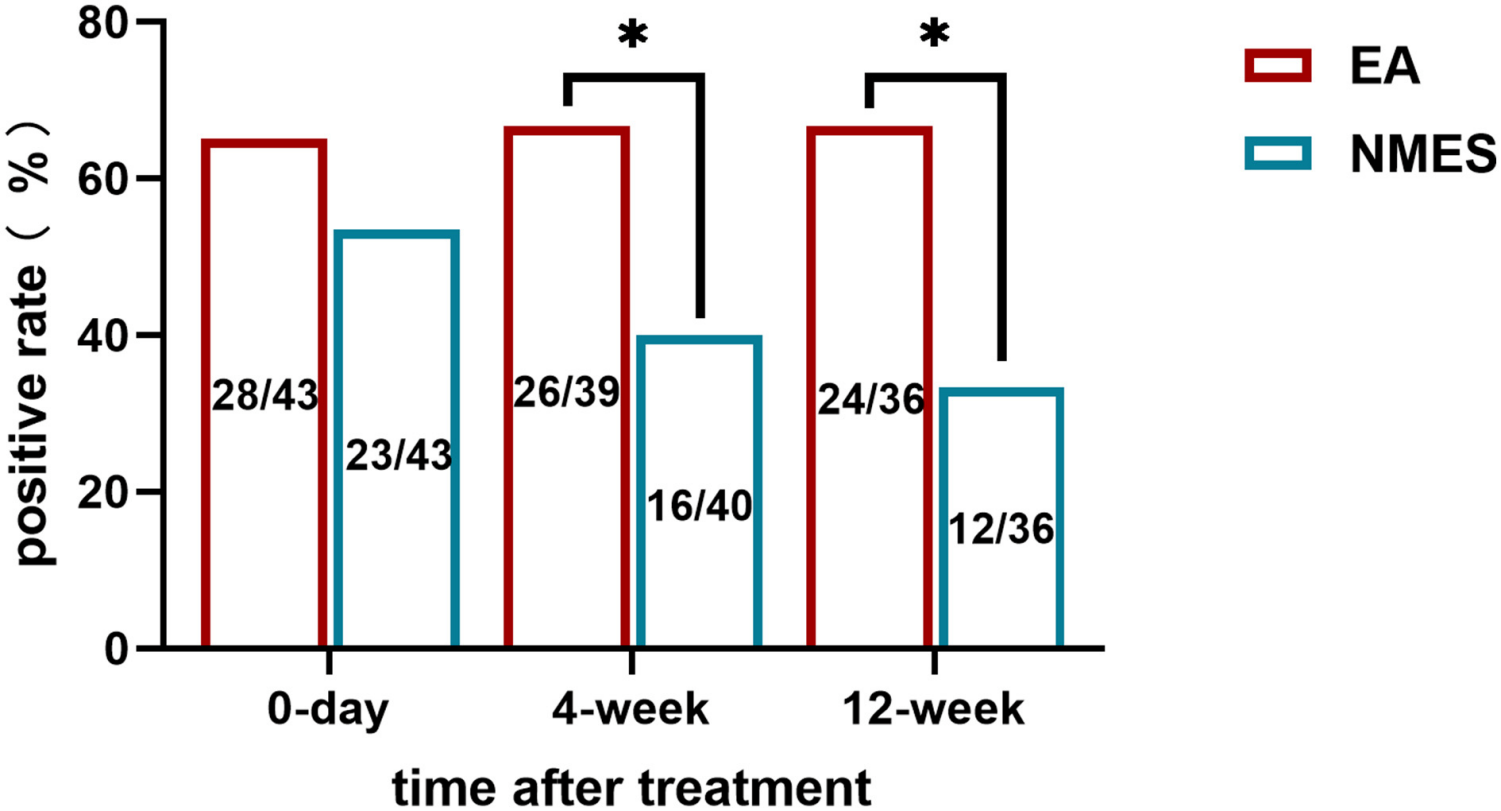
Rate of positive response to treatments in **(A)** immediately after treatment; **(B)** 4-weeks follow-up; and **(C)** 12-weeks follow-up.

**Figure 3:**
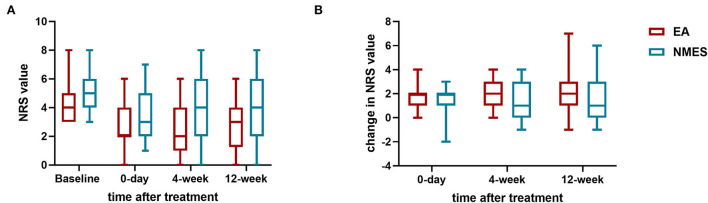
NRS values. **(A)** NRS values before and after treatments; **(B)** changes in NRS values from baseline.

For the primary outcome, no difference has been found in the rate of positive response in the assessment visit, with 65.1% (28 in 43) individuals reporting a positive response to EA treatment, while 53.5% (23 in 43) in the NMES. However, for secondary outcomes, significance presence in both of the 4-weeks (26 in 39, 66.7% vs. 16 in 40, 40%; *P* = 0.018) and 12-weeks (24 in 36, 66.7% vs. 12 in 36, 33.3%; *P* = 0.005) follow-up (Figure 2). Data of NRS values in different timepoints and change in NRS values to baseline are shown in Figure 3.

In the univariate logistic analysis, five variables were screened out as potential risk factors for positive treatment response with an unadjusted *P*-value <0.1, and there are treatment method, baseline VAS, heavy physical demand, baseline FABQ score, and history of the previous injury (Table 3) in the assessment visit and follow-ups. Further, we applied these five variables to a multivariable analysis, and the results suggest that in the assessment visit immediately after treatment, pain intensity [OR, 95% CI; 0.356 (0.133, 0.958), *P* = 0.041] and baseline FABQ score [OR, 95% CI; 1.057 (1.01, 1.107), *P* = 0.017] are associated with better treatment response, whereas their correlations disappeared in turn in the later follow-ups. EA is associated with better treatment response only 12 weeks later [OR, 95% CI; 4.045 (1.348, 12.137), *P* = 0.013, Table 4].

**Table 3:** Univariate analyses for treatment response.

**Variable**	**2-week**		**4-week**		**12-week**	
	**Hazard ratio** ** (95% CI)**	***P* value**	**Hazard ratio** ** (95% CI)**	***P* value**	**Hazard ratio** ** (95% CI)**	***P* value**
Treatment (EA vs. NMES)	2.645 (0.63, 11.108)	0.184	4.595 (1.108, 19.049)	0.036	8.031 (1.683, 38.312)	0.009
Baseline VAS moderate to	0.477 (0.196, 1.16)	0.100	0.336 (0.09, 1.252)	0.104	0.674 (0.181, 2.505)	0.556
Age (year)	0.847 (0.642, 1.117)	0.240	0.929 (0.71, 1.216)	0.592	1.066 (0.809, 1.406)	0.649
BMI	1.201 (0.907, 1.591)	0.200	0.923 (0.713, 1.195)	0.543	0.872 (0.661, 1.149)	0.330
Education background	2.621 (0.319, 21.523)	0.370	1.373 (0.162, 11.652)	0.772	0.423 (0.044, 4.064)	0.456
Serving time	3.311 (0.626, 17.52)	0.159	1.772 (0.393, 8.000)	0.457	1.109 (0.234, 5.251)	0.896
Smoking history	0.995 (0.986, 1.004)	0.274	0.747 (0.322, 1.729)	0.495	0.889 (0.360, 2.197)	0.799
Heavy physical demand	6.364 (0.977, 41.461)	0.053	2.275 (0.379, 13.644)	0.368	3.364 (0.575, 19.678)	0.178
History of injury	0.337 (0.091, 1.247)	0.103	1.591 (0.488, 5.184)	0.441	1.332 (0.390, 4.549)	0.648
Job type	0.963 (0.362, 2.561)	0.940	0.516 (0.186, 1.430)	0.203	0.500 (0.174, 1.432)	0.197
History of treatment for LBP	0.922 (0.175, 4.866)	0.924	0.423 (0.092, 1.950)	0.27	0.991 (0.214, 4.588)	0.991
Time of LBP	0.972 (0.219, 4.319)	0.970	1.167 (0.761, 2.225)	0.497	0.839 (0.198, 3.561)	0.812
Baseline ODI score	0.331 (0.026, 4.291)	0.398	0.441 (0.001, 3.944)	0.813	0.694 (0.001, 371.808)	0.909
Baseline FABQ score	1.037 (0.999, 1.077)	0.059	1.067 (0.994, 1.146)	0.071	1.002 (0.936, 1.072)	0.962
Symmetry of multifidus	2.73 (0.000, 12.026)	0.682	4.007 (0.000, 10.078)	0.888	3.918 (0.000, 13.496)	0.892
Contraction of multifidus	0.173 (0.000, 3.755)	0.939	3.407 (0.214, 54.262)	0.385	0.052 (0.000, 765.931)	0.731
CSA of multifidus	0.727 (0.139, 3.790)	0.705	2.301 (0.106, 12.005)	0.840	7.059 (0.000, 120.146)	0.590

EA, electroacupuncture; NMES, neuromuscular electrical stimulation; sd, standard deviation; LBP, low back pain; ODI, The Oswestry Disability Index; FABQ, fear-avoidance beliefs questionnaire; CSA, cross-sectional area.

**Table 4:** Multivariate analyses for risk factors associated with treatment response.

**Variable**	**2-week**		**4-week**		**12-week**	
	**Odd ratio (95% CI)**	***P* value**	**Odd ratio (95% CI)**	***P* value**	**Odd ratio (95% CI)**	***P* value**
Treatment (EA vs. NMES)	0.989 (0.390, 2.508)	0.981	2.494 (0.883, 7.044)	0.065	4.045 (1.348, 12.137)	0.013
Baseline VAS (moderate to severe pain vs. mild)	0.356 (0.133, 0.958)	0.041	0.405 (0.136, 1.206)	0.100	0.565 (0.192, 1.659)	0.299
Heavy physical demand	2.224 (0.745, 6.638)	0.152	2.413 (0.625, 9.316)	0.201	3.850 (1.003, 14.772)	0.049
History of injury	0.657 (0.260, 1.659)	0.374	1.391 (0.489, 3.954)	0.536	1.139 (0.395, 3.289)	0.810
Baseline FABQ score	1.057 (1.010, 1.107)	0.017	1.072 (1.017, 1.129)	0.009	1.022 (0.978, 1.068)	0.331

EA, electroacupuncture; NMES, neuromuscular electrical stimulation; FABQ, fear-avoidance beliefs questionnaire. Odds ratios are reported as a likelihood of positive treatment response with presence (for categorical variables) or each single unit increase (for continuous variables) in the specified variable, with ORs > 1 being associated with an increased likelihood of positive treatment response.

In linear regression analysis, six variables are included in the multivariable model after the previous univariate selection, namely, treatment method, baseline VAS score (continues), heavy physical demand, history of treatment for LBP, time of LBP, and baseline FABQ score (Supplementary Table 1). Baseline NRS score and a history of previous treatment for LBP seem to be highly correlated with decreased pain intensity in all follow-ups, while the treatment method is significantly correlated with decreased pain intensity only in the 4-weeks later [coefficient (95% CI), 0.861 (0.008, 1.713); *P* = 0.048, Supplementary Table 2].

**Figure 4:**
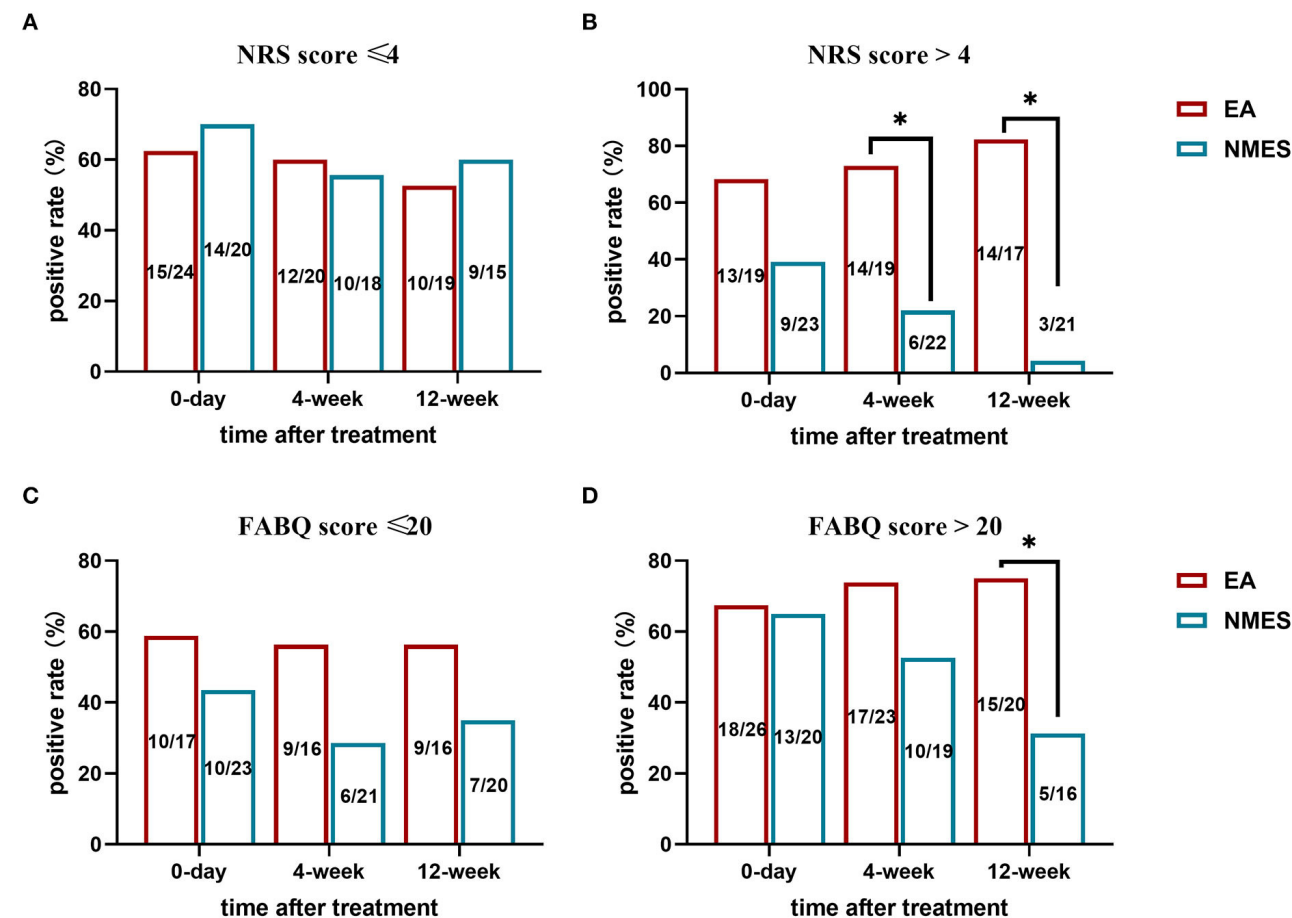
Subgroup analysis for the rate of positive treatment response in patients with **(A)** NRS score ≤4; **(B)** NRS score >4; **(C)** FABQ score ≤20; and **(D)** FABQ score >20. *P < 0.05.

Further, we conduct a subgroup analysis to compare the rate of positive treatment responses in patients with different baseline pain intensities and different baseline FABQ scores. It seems that while no difference has been found in patients with NRS score ≤ 4 or FABQ ≥20 (Figures 4A,C), EA treatment is associated with better long-term outcomes in LBP patients with severe pain scores (NRS score >4, Figure 4B) and stronger fear-avoidance beliefs (FABQ >20, Figure 4D).

## Discussion

The primary aim of this randomized clinical trial is to compare electroacupuncture with NMES for chronic LBP in active-duty navy populations. The second objective of the study is to investigate predictive factors for the treatment response. Our results indicate no significant superiority of electroacupuncture to physiotherapy in treating chronic LBP immediately after treatment. While in comparing long-term outcomes, EA is associated with a significant positive response to treatments in reducing pain intensity. Further, several factors which are potentially associated with treatment effects in short or long-term are identified, there are treatment method, baseline VAS, heavy physical demand, baseline FABQ score, and some more factors may associate with significant pain relief only in certain time points, including the history of previous injury, treatment for LBP, and time of LBP. Besides, by measuring thickness and CSA of multifidus before and after treatment *via* ultrasound, we find that both the two muscle electrical stimulation methods improve the multifidus function, which is in line with their analgesic effect on LBP. Interestingly, besides from prior history of LBP, we find none of the baseline characteristics nor the physical performance is associated with baseline back pain intensity.

The indication or superiority of these two methods has never been studied before, as it often causes confusion for military general physicians. Our results indicate no statistically significant difference between EA vs. NMES, in terms of the positive response rate and the changes in NRS values, immediately after a 2-weeks treatment diagram. However, we identify that EA may be associated with a longer analgesic effect for LBP 1 month or 3 months after treatment. Acupuncture has been used to treat pain for thousands of years in China, and is been even popular among military populations for its excellent safety profile and simplicity in implementation, even for medical professionals with no prior training. Further, it has been widely implemented at US Department of Defense and Veterans Affairs hospitals for treating acutely painful conditions in a variety of settings (27). A recent double-blind randomized clinical trial indicates that EA significantly improves several readiness outcomes of LBP (21). In term of NMES, results are controversial, and some studies report that it only accounts for 1.5–2 points decrease in pain intensity on an 11-point pain scale over the full treatment diagram, while some other study reports significant pain relief with sustained improvement for at least 4 months (28, 29). Our result is also in line with previous reviews to report that NMES treatment eases short-term musculoskeletal pain, while its long-term benefice is inconclusive.

Baseline pain score and FABQ score are two other factors associated with positive treatment response. Interestingly, in logistic regression, baseline pain intensity seems to be associated with immediate treatment effect; yet in the linear regression analysis, baseline NRS score is correlated with reduced pain score in almost all follow-ups. As a matter of fact, both of these variables are generally accepted as the main indicative symptoms and predictive factors in predicting treatment effects for chronic LBP, as higher NRS score indicates pain catastrophizing, and FABQ reflects the fear-avoidance beliefs (26). Fear-avoidance belief is a psychological assessment that details an individual's fear of pain and re-injury specific to LBP. Some studies even conclude that psychological factors are the strongest predictors of pain and disability in patients with cLBP (30). For this consideration, we further perform subgroup analysis for treatment outcomes in patients with different pain intensities or social mental states. It seems that patients with severe baseline NRS or FABQ scores might benefit more from electroacupuncture treatment. Note that the interpretation of these results on secondary outcomes should be tempered by the fact that the data of small samples are easily affected by certain outliers.

In this study, we also investigate the potential relations between muscular functions of multifidus and treatment outcomes. Symmetry, construction rate, and CSA of multifidus are estimated using the ultrasound technique. By comparing the muscular functions before and after treatment, we find a significant change in the symmetry and construction. However, none of these variables predicts treatment outcomes. Our results suggest that electoral therapy still affects the performance of multifidus; however, its treatment effect is far complicated than a mere improvement in construction strength or muscle mass. Actually, the function of muscles involved in core stabilization, such as the transversus abdominis, multifidi, and erector spinae muscles, has been identified as potential predictors of an individual's risk of developing chronic or recurrent LBP, and various prevention exercise programs for LBP have been rigorously studied in military population based on this theory (24). However, the mechanisms behind it remain elusive, and standardized exercise programs have not yet been identified. Noting that one more protective factor for treatment outcomes, including heavy physical demand, which may because of a fact that participant accepted this trial have more resting time during the treatment.

## Limitations

Several limitations of this study should be addressed. First, all participants were male soldiers who served in one naval unit, and the sample size is relatively small. Although this population provides convenience in randomization and follow-up owning to discipline among the servicemen, this may limit the application and explanation of other results, for instance, more analyses for change in multifidus functions during treatments seem to be an over-interpretation of our data. Second, blinding for the participants is not applied due to the nature of the treatments, and this may also cause significant information bias, as the main outcomes are based on the self-reported NRS score. Last but not least, several potential risk factors are not analyzed, including physical performance and phycological statues.

## Conclusion

According to our results, EA seems to be a more recommended treatment for military LBP, as both the EA and NMES are associated with a positive response in treating military cLBP, and EA exhibits lasting treatment outcomes in the later follow-ups. A lot of research is needed to explore efficient and standardized treatment sessions for military LBP and provide more information and evidence about indications for these treatments among young soldiers.

## Data availability statement

The raw data supporting the conclusions of this article will be made available by the authors, without undue reservation.

## Ethics statement

The studies involving human participants were reviewed and approved by Ethics and Security Committee of Eastern Hepatobiliary Surgical Hospital (EHBHKY2020-K-058). The patients/participants provided their written informed consent to participate in this study.

## Author contributions

Study concept and design: X-yM, X-lL, and F-xW. Drafting of the manuscript and analysis and interpretation of data: X-yM and J-yC. Follow-up and data collection: LB. Implement the trial: X-yM and Q-jL. Revision of the manuscript: X-lL and F-xW. All authors have read and approved the final manuscript.

## Conflict of interest

The authors declare that the research was conducted in the absence of any commercial or financial relationships that could be construed as a potential conflict of interest.

## Publisher's note

All claims expressed in this article are solely those of the authors and do not necessarily represent those of their affiliated organizations, or those of the publisher, the editors and the reviewers. Any product that may be evaluated in this article, or claim that may be made by its manufacturer, is not guaranteed or endorsed by the publisher.
