# Corrigendum: Comparative effectiveness of electroacupuncture VS neuromuscular electrical stimulation in the treatment of chronic low back pain in active-duty personals: A single-center, randomized control study

**DOI:** 10.3389/fneur.2022.1043063

**Published:** 2022-10-11

**Authors:** Xiao-yan Meng, Lan Bu, Jia-ying Chen, Qiu-jia Liu, Li Sun, Xiao-long Li, Fei-xiang Wu

**Affiliations:** ^1^Department of Critical Care Medicine, Eastern Hepatobiliary Surgery Hospital, Navel Medical University, Shanghai, China; ^2^Department of Anesthesiology and Pain Center, Shanghai Changhai Hospital, Navel Medical University, Shanghai, China; ^3^Department of Anesthesiology, Eastern Hepatobiliary Surgery Hospital, Navel Medical University, Shanghai, China; ^4^Department of Traditional Chinese Medicine, Shanghai Changhai Hospital, Navel Medical University, Shanghai, China; ^5^Department of Spinal Surgery, Shanghai Changhai Hospital, Navel Medical University, Shanghai, China

**Keywords:** neuromuscular electrical stimulation, chronic low back pain, military service, electroacupuncture, randomized control study

In the published article, there was an error regarding the affiliations for Lan Bu and Jia-ying Chen. They are not affiliated to Affiliation 1.

In the published article, there was an error in the author list, and authors Xiao-yan Meng, Lan Bu, Jia-ying Chen were erroneously excluded from sharing first authorship.

The authors apologize for this error and state that this does not change the scientific conclusions of the article in any way. The original article has been updated.

## Publisher's note

All claims expressed in this article are solely those of the authors and do not necessarily represent those of their affiliated organizations, or those of the publisher, the editors and the reviewers. Any product that may be evaluated in this article, or claim that may be made by its manufacturer, is not guaranteed or endorsed by the publisher.

