# Supplementary material for: Comparative effectiveness of electroacupuncture VS neuromuscular electrical stimulation in the treatment of chronic low back pain in active-duty personals: A single-center, randomized control study
Source: Front Neurol. 2022 Sep 13;13:945210. doi: 10.3389/fneur.2022.945210 (PMC9513143; doi:10.3389/fneur.2022.945210)
Supplement: Supplementary file 1 [file Data_Sheet_1.pdf]

Supplementary Table 1. Univariate linear regression analyses for changes in NRS.

Variable	2-week		4-week		12-week	
	Coefficient (95% CI)	P value	Coefficient (95% CI)	P value	Coefficient (95% CI)	P value
Treatment (EA vs. NMES)	0.348 (-0.019, 0.715)	0.063	0.312 (0.266, 1.484)	0.005	0.803 (0.038, 1.567)	0.041
Baseline VAS	0.107 (-0.018, 0.232)	0.092	-0.104 (-0.31, 0.116)	0.366	0.03 (-0.232, 0.292)	0.821
Age (year)	-0.075 (-0.089, 0.062)	0.724	0.052 (-0.053, 0.085)	0.651	-0.194 (-0.139, 0.018)	0.128
BMI	0.024 (-0.073, 0.09)	0.835	-0.025 (-0.106, 0.085)	0.827	-0.033 (-0.185, 0.142)	0.797
Education background	0.155 (-0.243, 0.937)	0.244	0.02 (-0.782, 0.936)	0.859	-0.004 (-1.057, 1.027)	0.977
Serving time	0.216 (-0.182, 0.664)	0.260	0.014 (-0.375, 0.423)	0.906	-0.088 (-0.584, 0.407)	0.723
Smoking history	-0.102 (-0.004, 0.001)	0.353	0.078 (-0.281, 0.576)	0.496	0.174 (-0.163, 0.89)	0.173
Heavy physical demand	0.334 (-0.12, 0.789)	0.148	0.192 (-0.115, 1.48)	0.092	0.951 (-0.002, 1.905)	0.050
History of injury	-0.18 (-0.568, 0.209)	0.360	-0.027 (-0.747, 0.587)	0.812	0.043 (-0.664, 0.932)	0.738
Job type	0.003 (-0.285, 0.293)	0.980	0.054 (-0.336, 0.544)	0.639	0.121 (-0.252, 0.71)	0.345
History of treatment for LBP	-0.334 (-0.607, -0.061)	0.017	-0.041 (-0.79, 0.55)	0.723	-0.186 (-1.496, 0.222)	0.143
Time of LBP	-0.109 (-0.218, -0.001)	0.048	0.086 (-0.403, 0.894)	0.453	-0.012 (-0.802, 0.73)	0.926
Baseline ODI score	-0.091 (-0.122, 0.051)	0.414	-0.044 (-0.171, 0.116)	0.703	0.044 (-1.847, 2.615)	0.732
Baseline FABQ score	0.028 (0.015, 0.041)	0.000	0.23 (0.001, 0.047)	0.043	0.025 (-0.004, 0.054)	0.086
Symmetry of multifidus	0.162 (-1.605, 4.236)	0.371	0.104 (-1.954, 5.189)	0.37	-0.116 (-5.64, 2.118)	0.367
Contraction of multifidus	-0.104 (-11.407, 6.459)	0.582	0.079 (-6.737, 13.54)	0.506	-0.174 (-17.472, 3.411)	0.183
CSA of multifidus	-0.075 (-1.031, 0.566)	0.562	0.077 (-0.791, 1.59)	0.506	0.008 (-1.346, 1.434)	0.95

Abbreviation: EA, electroacupuncture; NMES, neuromuscular electrical stimulation; sd, standard deviation; LBP, low back pain; ODI, The Oswestry Disability Index; FABQ, fear-avoidance beliefs questionnaire; CSA, cross-sectional area.

Supplementary Table 2. Multivariate linear regression analyses for changes in NRS.

Variable	2-week		4-week		12-week	
	Coefficient (95% CI)	P value	Coefficient (95% CI)	P value	Coefficient (95% CI)	P value
Treatment (EA vs. NMES)	0.466 (-0.245, 1.177)	0.196	**0.861 (0.008, 1.713)**	**0.048**	0.668 (-0.512, 1.849)	0.263
Baseline VAS	0.323 (-0.037, 0.684)	0.078	0.936 (0.332, 1.54)	0.003	0.875 (0.053, 1.698)	0.037
Heavy physical demand	0.054 (-0.064, 0.171)	0.366	-0.629 (-1.218, -0.039)	0.037	-0.423 (-1.183, 0.338)	0.272
History of treatment for LBP	**0.465 (0.039, 0.891)**	**0.033**	0.715 (-0.03, 1.461)	0.060	**0.936 (0.007, 1.866)**	**0.048**
Time of LBP	-0.271 (-0.663, 0.12)	0.171	-0.43 (-1.077, 0.218)	0.190	-0.582 (-1.418, 0.253)	0.169
Baseline FABQ score	-0.036 (-0.192, 0.119)	0.643	0.255 (-0.359, 0.868)	0.411	-0.244 (-1.023, 0.536)	0.535

Abbreviation: EA, electroacupuncture; NMES, neuromuscular electrical stimulation; LBP, low back pain; ODI, The Oswestry Disability Index; FABQ, fear-avoidance beliefs questionnaire; CSA, cross-sectional area.
