# Supplementary material for: Comparative effectiveness of electroacupuncture VS neuromuscular electrical stimulation in the treatment of chronic low back pain in active-duty personals: A single-center, randomized control study
Source: Front Neurol. 2022 Sep 13;13:945210. doi: 10.3389/fneur.2022.945210 (PMC9513143; doi:10.3389/fneur.2022.945210)
Supplement: Supplementary file 2 [file Table_1.DOC]

| **Brief Questionnaire for Military LBP**  ID | | |
| --- | --- | --- |
| Name |  | Age |
| Gender |  |  |
| Height cm | | Weight kg |
| Education background | | Service time |
| Phone number | | |
| Smoking history | | |
| Type of troops | | |
| Low back pain in last week： | | |
| With leg pain □ no □ yes | | |
| Time for low back pain | | |
| History of spinal injury □ no □ yes | | |
| Pain score (0-10 points） | | |
| 0（no pain）、10（highest level of pain），mark pain intensity with × on the line below. | | |
| |  | | --- | | | |
| 1. 10   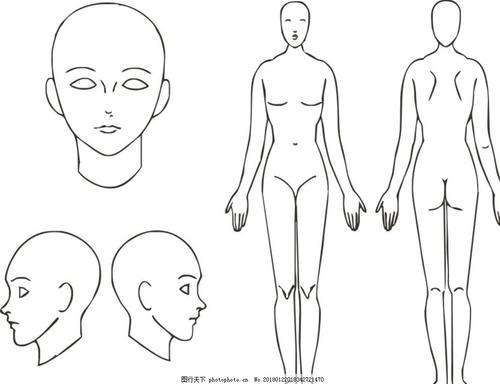  if you have a pain episode  in the last week, please  draw it in this picture. | | |

Data of filling:
