# Supplementary material for: Comparative effectiveness of electroacupuncture VS neuromuscular electrical stimulation in the treatment of chronic low back pain in active-duty personals: A single-center, randomized control study
Source: Front Neurol. 2022 Sep 13;13:945210. doi: 10.3389/fneur.2022.945210 (PMC9513143; doi:10.3389/fneur.2022.945210)

**The detailed treatment protocols**


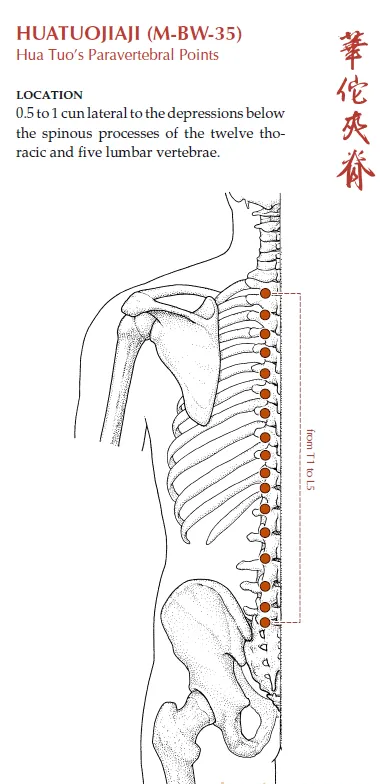
For participants assigned to the electroacupuncture group, the acupuncture procedures were performed in accordance with the Standards for Reporting Interventions in Clinical Trials of Acupuncture (STRICTA) guidelines. The participant was positioned comfortably in the prone position. After skin degerming, acupuncture needles are inserted into the skin of bilateral JiaJi (M-BW-35) acupoints (L3-S1) for about 5 cm, according to the World Health Organization Standardized Acupuncture Point Location, which is located in the lumbar region 2-3 cm lateral to the posterior median line. then, the acupuncturist manipulated the needle with a lifting, thrusting, and twirling maneuver until feelings of soreness and distension were felt and radiated to the hips and lower limbs. after all needles were placed, the electric apparatus was applied to the acupoints with a dilatational wave using a 50 Hz frequency and a comfortably tolerated maximum current intensity.

We used huatuo Brand stainless steel needle for acupuncture (0.3 × 100 mm, Suzhou Medical Appliance Factory in China), and a G6805-2 electric stimulator (Shanghai Huayi Medical Instrument in China Co., Led) in the treatment.

For patients assigned to neuromuscular electrical simulation group, 2 pairs of 40 × 40 mm electrodes were placed on their back, which is at the same acupoints to the electroacupuncture. The electrotherapy apparatus (Xiangyu Medical Equipment in China Co., Led) was turned on and we select a medium burst frequency of 1 KHz. The current intensity was gradually increased to the maximum tolerance of participants. Each participant treated for a total of 30 minutes each session, for 6-7 times in 2 weeks.

**Ultrasound imaging assessment for multifidus**

Ultrasound scanning of the muscles was carried out using an ultrasound system (SonoSite, USA). Multifidus muscle thickness (cm) and CSAs (cm2) are measured in the B scan mode. An HST/10-5 MHz 25 mm linear probe was used to image the surfaces of the muscles. During the study, subjects were posed face down in a relaxed, neutral head position, with their arms relaxed at their sides. A small pillow was placed under the stomach to reduce lordosis of the lumbar part of the spine. Ultrasound scanning of multiple muscles was performed in parallel on both sides of the spine in the region of the L4–L5 lumbar segments. The fourth lumbar vertebra (L4) is determined by palpation, starting from the wings of the hip bones towards the center line. Data of multifidus is measured in rest state at first, then participants are asked to have back extension to their greatest effort, then data of multifidus at contraction state is measured as well. Symmetry of multifidus is defined of proportional difference of relatively larger side to smaller side at rest state. Contraction of multifidus is defined as proportional difference of mean thickness at contraction to rest state. All assessments were conducted by the same investigator in the same place, to reduce measurement errors


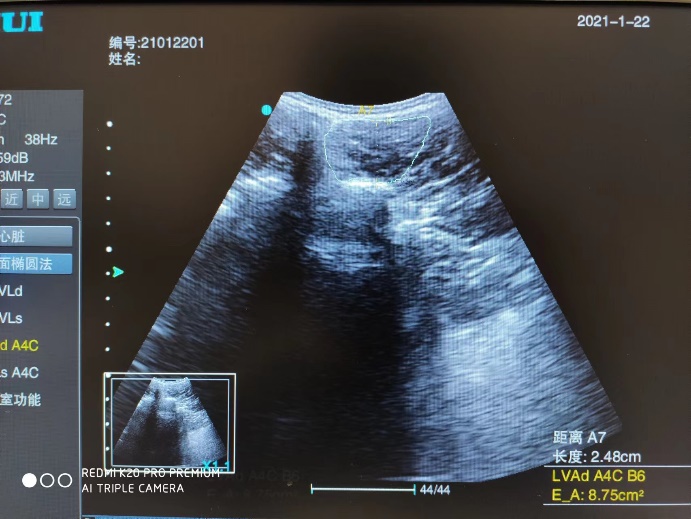

Supplement: Supplementary file 3 [file Table_2.DOCX]
